# Site-selective lysine conjugation methods and applications towards antibody–drug conjugates

**DOI:** 10.1039/d1cc03976h

**Published:** 2021-09-27

**Authors:** Muhammed Haque, Nafsika Forte, James R. Baker

**Affiliations:** Department of Chemistry, University College London 20 Gordon Street London WC1H 0AJ UK j.r.baker@ucl.ac.uk

## Abstract

Site-selective protein modification is of significant interest in chemical biology research, with lysine residues representing a particularly challenging target. Whilst lysines are popular for bioconjugation, due to their nucleophilicity, solvent accessibility and the stability of the resultant conjugates, their high abundance means site-selectivity is very difficult to achieve. Antibody–drug conjugates (ADCs) present a powerful therapeutic application of protein modification, and have often relied extensively upon lysine bioconjugation for their synthesis. Here we discuss advances in methodologies for achieving site-selective lysine modification, particularly within the context of antibody conjugate construction, including the cysteine-to-lysine transfer (CLT) protocol which we have recently reported.

## Abstract 2

In this feature article we discuss developments in site-selective lysine modification methodologies and their application towards the synthesis of antibody–drug conjugates; including our recent work on a cysteine-to-lysine transfer (CLT) protocol.

## Graphical abstract

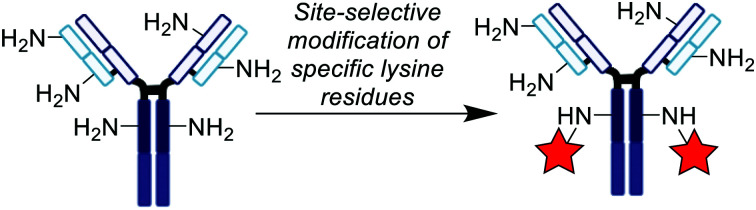

## 1. Introduction

Protein modification is of significant importance in both natural processes and in chemical biology research. In nature, post-translational modifications (PTMs) are crucial in enabling proteins to exhibit incredible structural and functional diversity.^1^ In chemical biology, protein modifications allow the generation of diverse bioconjugates, with immense therapeutic and diagnostic potential. One particularly interesting class of therapeutics for anti-cancer purposes are antibody–drug conjugates (ADCs), which combine the specificity of a monoclonal antibody (mAb) for its target antigen with the potency of an attached cytotoxic drug.^2^ mAbs are Y-shaped glycoproteins composed of two light-chains (LC) and two heavy-chains (HC). Individual LCs and HCs are connected by a disulfide bond, forming a half-antibody, which is then linked to another half-antibody through inter-chain disulfide bonds in the hinge region of the HCs. Non-covalent interactions between the individual chains also help maintain the structure and stability of mAbs.^3^ There are six complementarity-determining regions (CDRs) in the variable domains responsible for the specificity of antibodies towards diseased cells. Whilst nature is capable of utilising its diverse array of enzymes to achieve complete chemo- and site-selectivity in the modification of such proteins, current chemical biology protocols to achieve the same feat are an area of extensive research. Limitations in bioconjugation methodologies have impacted the success and clinical potential of the current generation of ADCs. For example, heterogeneity is a significant concern, where the drug-to-antibody ratio (DAR) varies within a single mixture, as does the precise site of conjugation on the antibody. This has contributed to poorer clinical outcomes, affecting pharmacokinetics, efficacy, and safety profiles. For example, Mylotarg, the first ADC approved, is a lysine conjugated ADC, and was temporarily withdrawn from the market due to its significant off-site toxicity; caused in part by its heterogeneity and early payload loss.^4^ Interestingly, in specific applications, ADCs heterogeneously labelled at lysine have been shown to demonstrate superior activity to homogeneous cysteine-conjugated counterparts.^5^ However, Lambert *et al.* conclude in this work that the complexity of ADCs and cancer as a disease means it is difficult to define a simple framework to follow for ADC design. It is necessary to thoroughly investigate conjugation chemistry, payload type, and target antigen – understanding the interplay between these factors is crucial and may vary in different cancer types. Nonetheless, it is still widely understood that improving homogeneity of ADCs can improve pharmacokinetics and provide better therapeutic indexes that ultimately translate to better clinical outcomes.^6^

Thus, there is a pressing need for the development of novel chemical strategies to achieve chemo- and site-selective modifications of antibodies for the synthesis of next-generation ADCs that are characterised by superior *in vivo* outcomes. The most commonly targeted residues for protein modifications are cysteine and lysine. The former due to its low relative abundance and high nucleophilicity at physiological pH; however, its involvement in disulfide bonds plays a critical role in maintaining protein structural integrity and stability, and the lack of easily accessible residues means cysteine bioconjugation is not always favourable. Mutagenesis can be employed to insert free cysteine residues, though this results in more technical complexity,^7^ and there is a requirement to control the oxidation state of the thiol group. For further information on cysteine bioconjugation methods, readers are referred to other reviews in this area.^8–10^ The latter, lysine, has been extensively studied in many bioconjugation applications, including in the synthesis of ADCs,^11–13^ drug delivery,^14^ and antimicrobial vaccines.^15^ Lysine is a nucleophilic amino acid with an ε-amino sidechain (p*K*_a_ ∼ 10.5), which is predominately positively charged at physiological pH, and found in high abundance on the surface of proteins; making lysine the most conventional and facile target for modification. The high lysine abundance, however, presents a technical challenge – controlling chemoselectivity and regioselectivity of these modifications is not a simple task, and thus heterogeneity is common.

**Scheme 1:**
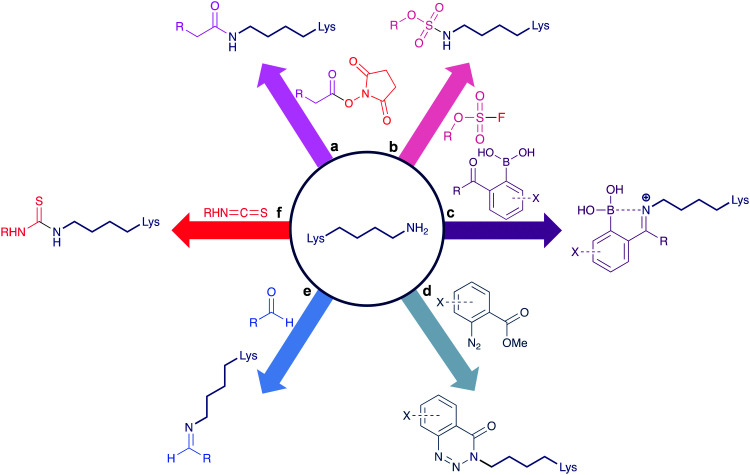
Conventional lysine bioconjugation reagents. (a) NHS esters,^16^ (b) sulfonyl fluorides,^21,22^ (c) iminoboronates,^23,24^ (d) diazonium salts,^25^ (e) aldehydes,^26,27^ (f) isothiocyanates.^28^

Conventional reagents for the modification of lysine residues include *N*-hydroxysuccinimide (NHS) esters (Scheme 1a).^16^ These are highly reactive, commercially available reagents that contain an activated acyl group. Capable of forming amide bonds between pH 7.0–9.0 in short timeframes, NHS esters have proven to be the most popular lysine modification reagent, with the first generation of ADCs utilising this technology for their synthesis. However, the drawback of employing such reagents is the resultant ADC heterogeneity. Kadcyla, a HER2-targeting ADC composed of the mAb trastuzumab and the cytotoxin DM1, for example, features mixtures of species with DARs varying between 0–8,^17^ and in a typical ADC this translates to ∼40 different lysines being modified.^18^ NHS esters can also suffer from hydrolysis and chemoselectivity issues, with competing attachments to residues including histidine, serine, threonine, and tyrosine.^19,20^ Other reagents (Scheme 1b–f) have sought to improve the chemoselectivity issues of NHS esters, including the sulfonyl halides,^21,22^ iminoboronates,^23,24^ diazonium salts,^25^ aldehydes,^26,27^ and isothiocyanates,^28^ though most of these strategies have not been explored for antibody modification.

Site-selectivity remains a challenge that needs to be addressed in order to develop robust next-generation lysine conjugates. Promisingly, recent strategies have demonstrated a capacity for overcoming previous shortcomings through novel chemistry and reagents. In this review article, we will discuss the three most prominent categories – direct modification of the most reactive lysine residue; enzymatic approaches; and proximity-induced modification. We will predominantly be focusing on methods which have already been applied in the context of antibody conjugation. In this last section we will include a discussion of our recent contribution, a cysteine-to-lysine transfer (CLT) protocol, which enables sites-selective modification of lysine residues on a Fab antibody fragment, generating highly homogeneous lysine conjugates.

## 2. Selective modification of the most reactive lysine residue

**Scheme 2:**
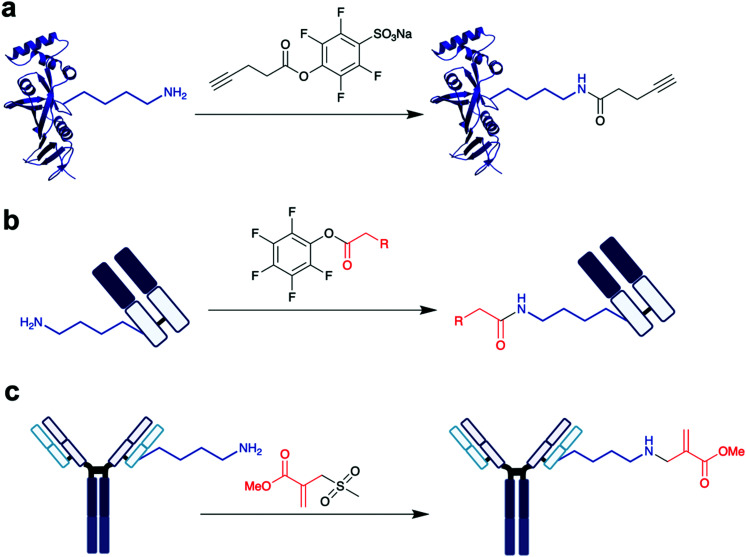
Examples of different methodologies for the labelling of the most reactive lysine. (a) STP used by Cravatt *et al.* for the investigation of global reactive lysines.^29^ PDB 3HY8 (b) Cellitti *et al.*'s fluorophenyl ester reagents, used for the labelling of K188 on the LC of Fab.^32^ (c) Sulfonyl acrylate modification of lysine on trastuzumab, by Bernardes *et al.*^36^

Individual lysine residues within a protein can have significantly different reactivity towards reagents, due to solvent accessibility, higher-order structural features and the surrounding protein environment. Cravatt *et al.* reported a chemical proteomic platform for the quantification of reactive lysine residues in native biological systems.^29^ In total, they quantified 9000 lysine residues and found 100 lysines of superior reactivity located within protein pockets – using lysine-reactive sulfotetrafluorophenyl (STP) electrophiles, they could selectively bind to these hyper-reactive lysines (Scheme 2a). Targeting such uniquely reactive lysines has thus become quite a popular method of achieving some site-selective modification on certain specific proteins.^30^

In 2012, Weil *et al.* site-selectively modified a lysine residue, K1, on RNase A; which represents a good example of the opportunities, and associated challenges, when attempting to achieve kinetic control in lysine modification.^31^ Using a biotin NHS ester at excess equivalents, they experienced a large distribution of lysine additions, typical of any NHS ester bioconjugation. By lowering equivalents of the biotin NHS ester to 1 equivalent, while the distribution of lysine additions was decreased with the major product now monobiotinylated RNase A, some double and triple additions were still observed. Further decreasing the equivalents to 0.5 equivalents assisted in further promoting exclusive single lysine addition, though in an incomplete manner. Weil *et al.* determined that careful control and addition of the NHS ester can further reduce excess modifications – adding 0.01 equivalents over 100 min (totaling 0.5 equivalents) yielded the monobiotinylated RNase A modified at K1, in a 92% yield based on recovered unmodified protein. Even slight increases in equivalents resulted in heterogeneity. For the same reason, an overnight quench of unreacted biotin NHS ester using ethanolamine was shown to be necessary to prevent excess addition. The same conditions were employed for the site-selective modification of lysozyme C and somatostatin, with lysine modified at K1 and K9 respectively. Weil *et al.* used their developed protocol to incorporate an alkyne handle on K1 of RNase A, using copper-catalysed azide–alkyne cycloaddition (CuAAC) to attach coumarin. The strictly controlled conditions and incomplete conversions necessary to achieve homogenous lysine bioconjugation likely limits the applicability of this method to certain peptides and proteins which contain relatively few lysine residues, as significantly more side-reactions are possible on lysine-rich proteins such as mAbs. Chemoselective limitations of NHS esters also means other nucleophilic residues could unfavourably influence modification sites.

Utilising the protein environment surrounding the targeted lysine residue can be a means to achieving site-selective modification, as this environment can confer unique properties onto the lysine. Cellitti *et al.* discovered that nearby histidine and aspartic acid residues can help accelerate rates of lysine labelling relative to ∼40 other lysines in an antigen-binding fragment (Fab) of the humanised mAb trastuzumab.^32^ To achieve this, fluorophenyl esters were used (Scheme 2b), as well as NHS esters for comparison. The group demonstrated that this fluorophenyl ester reagent only undergoes Fab addition when K188 exists – point mutation to arginine (K188R) prevented any bioconjugation. D151 and H189 were also shown to be essential for K188 labelling, as no modification was observed when either D151 or H189 were mutated into alanine. Fluorophenyl esters were shown to be far more selective than NHS esters, as the latter resulted in varying extents and distributions of Fab labelling. Various chemical cargos were explored, and each fluorophenyl ester derivative retained the same number and distribution of modifications. Using a fluorophenyl biotin reagent, ∼75% (at RT) to ∼80% (at 4 °C) conversion to a labelled K188 Fab conjugate was achieved. However, small amounts of multiple additions on the LC and HC were observed, even at 4 °C. Utilising flow chemistry procedures helped to further improve these selectivity issues, with complete conversion to the singly modified K188 Fab conjugate. In contrast, the NHS ester derivative continued to provide heterogeneous mixtures. Therefore, it is evident that adjusting reaction setups could help improve the homogeneity of synthesised bioconjugates. Flow chemistry is increasingly popular in small molecule synthesis, as it enables more precise control of reagent additions and stoichiometries.^33^ More recently, its benefits have been utilised for the synthesis of bioconjugates.^34,35^

Bernardes *et al.* investigated the use of sulfonyl acrylate reagents for selective antibody conjugation.^36^ Computer-assisted reagent design helped the group select an appropriately structured sulfonyl acrylate (Scheme 2c), with identification of an intermolecular hydrogen bond between this acrylate and the lysine side-chain in a chair-like conformation found to be key for desirable reactivity. With this knowledge, the use of this acrylate was explored on the mAb trastuzumab. A selective addition to a lysine on the LC was observed, after 2 h at 37 °C, pH 8.0. HER2 affinity was retained, indicating that the modification does not occur in the CDRs. It is worth noting that no conjugate digestion and sequencing was reported, thus while it is evident conjugation occurs on the LC, the level of residue-selectivity is not determined. Sequencing was conducted on the other proteins studied such as lysozyme, C2Am, and Annexin V, where significant selectivity was observed. Promisingly, the sulfonate acrylate reagent did not react with the thiol of free cysteine residues in either C2Am or Annexin V. These sulfonyl acrylates thus offer promising avenues to dual functionalisation of the studied proteins, where cysteine and lysine residues can be modified by different classes of reagents.

**Scheme 3:**
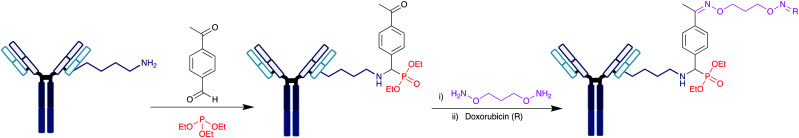
Rai *et al.*'s work on aldehydes as reagents for lysine modification on an antibody, utilising a phospha-Mannich protocol, followed by subsequent functionalisation of an aldehyde using a functionalised hydroxylamine. R = doxorubicin.^39^

Extensive work on lysine modification using aldehydes has been conducted by Rai *et al.*, with these investigations demonstrating selectivity for single lysine residues, albeit with incomplete conversions.^37,38^ A multicomponent approach was recently reported which yielded promising results, utilising a phospha-Mannich reaction (Scheme 3).^39^ On multiple small proteins, it was possible to achieve site-selective modification of a single lysine residue with an aldehyde, forming an imine as an *in situ* electrophile. Irreversible chemoselective substitution at the imine occurred using nucleophilic alkylphosphites, generating stable, site-selectively modified products. Late-stage functionalisation of their multicomponent reagent was also possible, using hydroxylamine derivatives. Low conversions of 32–41% within 0.5–10 h were obtained on proteins such as ubiquitin, RNase A, and cytochrome *C*. Rai and co-workers next explored the viability of the strategy for ADC synthesis. On trastuzumab Fab, they were able to demonstrate site-selective modification of K183, though the maximum conversion was low, at 27% after 6 h. Further work on trastuzumab itself could generate an ADC loaded with a doxorubicin (Dox) cytotoxin – a multi-step process obtained trastuzumab–Dox with a low average DAR of 0.92 after 22 h total reaction time, at 25 °C, pH 7.8. Cell assay studies of this ADC did confer HER2 selectivity in HER2+ SKBR-3 cells that exceeds that of unconjugated doxorubicin, with greater cytotoxicity than unmodified trastuzumab. However, low DARs are generally unfavourable for effective ADCs *in vivo*.^40^

**Scheme 4:**
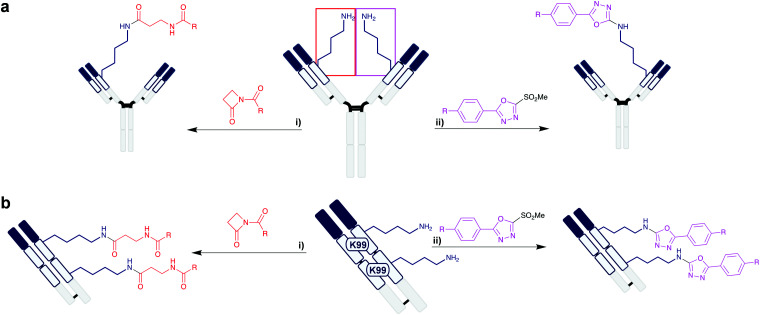
Rader *et al.*'s investigations on multi-domain antibody structures, utilising the K99 residue of the h38C2 domain. (a) Modification of K99 on the DVD, consisting of full mAb. (i) Lactam reagent.^42^ (ii) Methylsulfonyl reagent.^43^ R = MMAF. (b) Modification of two lysine residues on the TVD, consisting of Fab. (i) Lactam reagent. (ii) Methylsulfonyl reagent. R = MMAF.^44^

Lysine residues located in deep hydrophobic pockets can have significantly lowered p*K*_a_s and hence be suitable sites for selective modification. The humanised anti-hapten catalytic mAb h38C2 contains a very deep 11 Å hydrophobic pocket containing a native lysine (K99) with a p*K*_a_ of 6.0.^41^ Thus, it is possible to achieve selective modification of K99. Rader *et al.* engineered a dual-variable domain (DVD) by combining the variable regions of h38C2, which contains the reactive lysine, and trastuzumab (Scheme 4a).^42^ Attachment of the cytotoxin monomethyl auristatin F (MMAF) at the lysine was achieved selectivity using a β-lactam handle, which formed a stable amide bond at pH 7.4 in 4 h at 22 °C. The generated ADC was highly homogeneous, with all loading occurring on the HC K99 residue, and with a DAR of 2.0. The resultant anti-HER2 ADC demonstrated better tumour regression and survival than the clinically approved Kadcyla at the same dosage. Additionally, ADCs synthesised through this methodology were then used to target the antigens CD183 and CD79B, which are overexpressed in multiple myeloma and non-Hodgkin lymphoma respectively, demonstrating IC_50_ values in the sub-nanomolar range. Rader *et al.* further demonstrated K99 to be a more broadly applicable modification site, as they also explored the use of heteroaryl methylsulfonyl reagents for lysine labelling on h38C2.^43^ These reagents resulted in stable lysine modification through a selective arylation reaction in 4 h at 22 °C, pH 7.4. Compared to β-lactam reagents, the methylsulfonyls can be synthesised more readily, using commercially available reagents on solid-phase resins without the necessity of purification. Recognising that the DVD-ADCs may be unsuitable for the treatment of solid cancers due to their large molecular weight (∼200 kDa), the group also employed their β-lactam and methylsulfonyl reagents on engineered triple variable domains (TVDs) based on Fab, which consist of two h38C2 regions and one anti-HER2 antigen recognition site, providing a Fab conjugate with DAR 2.0 at a lower molecular weight of ∼100 kDa (Scheme 4b).^44^ Homogeneity was again observed in the resultant TVD–Fab conjugate, site-selectively attaching the cytotoxic payload at K99 of both h38C2 domains, with similar activity against HER2+ cancer cells to that of the DVD work – the reduced size of the TVD improved tumour site accumulation, compared to the larger DVDs previously synthesised by the group.

It is evident that achieving the selective modification of the most reactive lysine in a protein is a challenging endeavour, which is commonly limited to simpler proteins which do not contain multiple residues competing for the chemical reagent. The significant benefit of such an approach when applicable is that commercially available and readily accessible reagents are effective, and the bioconjugation protocol itself is straightforward. However, larger proteins, including antibodies, are unlikely to undergo completely selective modification unless a specific lysine with increased reactivity is identified. Additionally, site-selective residue modification through targeting only the most reactive lysine has its limitations and does not address more complex functionalisation challenges in lysine bioconjugation; it requires a specific protein environment to provide a lysine of distinctive reactivity, and it is not always possible to choose the site of conjugation to favour a residue that avoids interrupting biochemical functionality.

## 3. Enzyme-directed modification

Enzymes can provide exquisite site-selectivity and specificity under biologically compatible conditions, due to their highly ordered binding sites. Much work has been done on utilising enzymatic reactions for bioconjugation, for the purposes of labelling,^45,46^ ADC synthesis,^47^ and other commercial uses.^48^

**Scheme 5:**
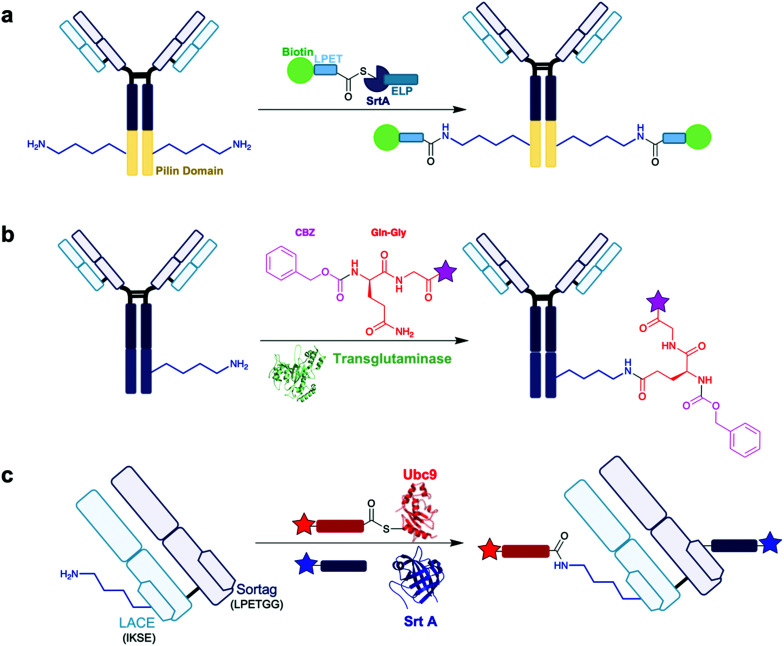
Examples of enzymatic site-selective modification. Star = functionality. (a) Lysine modification on a pilin domain inserted within a mAb, using the Srt A enzyme as explored by Chilkoti *et al.*^49^ (b) Transglutaminase-based strategy for lysine modification of K447 on IgG, utilised by Kline and co-workers for the synthesis of functionalised antibody conjugates. Pink star = biotin, azide, PEG_*n*_–BCN or PEG_2_AuF.^54^ PDB 1IU4. (c) LACE method for lysine modification using Ubc9 (PDB 1A3S) to recognise a IKSE sequence on the LC of Fab, and Srt A (PDB 1T2W) to recognise a LPETGG sequence on the HC of Fab, by Bode and co-workers. Red star = rhodamine, blue star = coumarin.^56^

An example of a lysine-targeting enzyme is sortase A (Srt A) from *Staphylococcus aureus*, which catalyses the covalent ligation between two amino acids *via* an isopeptide bond. Srt A recognises the primary acid sequence LPXTG – it first cleaves the amide bond between threonine and glycine, undergoing a thioesterification with its active site cysteine and the carbonyl group of threonine. This thioester can be substituted by an amino group to form a stable amide bond. It is possible to use this activity to achieve site-specific modification of a range of proteins, as reported by Chilkoti and co-workers (Scheme 5a).^49^ Initial work on recombinant proteins containing pilin domain peptides provided promise as a strategy for site-selective lysine modification. These pilin domain peptides typically contain a single lysine residue, and Chilkoti *et al.* could demonstrate modification of this lysine using Srt A and small molecule containing a LPXTG recognition peptide – in these initial studies, site-selective modification occurred in a 75% conversion rate. They then progressed onto the anti-HER2 mAb 4D5 for the synthesis of an antibody conjugate. Importantly for their strategy, 4D5 contains a pilin domain at the carboxyl terminal of the HC. Incubation of 4D5 with Srt A and a biotin-LPETGRAGG peptide overnight demonstrated site-selective modification of the lysine residues located only on the inserted pilin domains, with no labelling observed elsewhere on the mAb, yielding an antibody conjugate with a biotin–antibody-ratio of 1.8 in 90% conversion. The resultant lysine conjugate demonstrates impressive homogeneity. More recently, further work by Clubb *et al.* found a *Corynebacterium diphtheriae* Srt A analogue can improve transpeptidation activity by a factor of 7-fold whilst retaining site-selectivity.^50^ It is evident from these studies that Srt A is a very attractive and capable enzyme for lysine modification. Indeed, there are other notable examples exploring application of Srt A for antibody conjugation.^51–53^

Another enzyme used for ADC synthesis in enzymatic approaches is microbial transglutaminase (MTG), which catalyses the formation of amide bonds between the γ-carboxyamide group of glutamine and the ε-amine group of lysine. This occurs by initial thioesterification, by attack of the active site cysteine on the γ-carboxyamide and loss of ammonia; and finally amide formation by reaction with the primary amine of the lysine. MTG is a cheap, readily available enzyme that functions under a diverse range of different temperatures, salt concentrations, and pH. Kline and co-workers reported an approach that could utilise MTG into targeting lysine residues as acyl acceptors on a mAb (Scheme 5b).^54^ The group recognised that while the C-terminal codon of an IgG is a lysine (HC K447), serum-derived and recombinantly-expressed IgGs are however missing this lysine residue, due to cleavage by carboxypeptidase B in the production host. None of the native lysine residues in the LC or HC are normally transamidated by MTG, however the group were interested in understanding whether the normally absent K447 residue could be a potential site of modification. K447 is located within a solvent-exposed loop – acyl acceptor sites in flexible loops are found to be preferred sites of MTG activity.^55^ Thus, the group first synthesised an IgG where K447 could be conserved as a potential acyl acceptor in the flexible loop, through a sequence extension with a single leucine. This prevented K447 cleavage during IgG expression and enabled MTG-mediated K447 transamidation upon addition of ZQG-biotin in 16 h at 37 °C. The group also conducted a widespread mutagenesis study where they investigated point mutations into lysines to locate other potential sites of MTG transamidation – many suitable sites for transamidation were detected, mostly located in the same exposed flexible loop domain as K447. Thus, it is clear MTG can denote significant selectivity towards this HC loop region. An ADC was then synthesised through this protocol – they first produced ZQG derivatives containing an azide, alkyne, or linker-cytotoxin moieties. The various mAb mutants synthesised by the group produced results with differing DARs. In the mAb species where a leucine residue is added after K447, DARs of 1.8, 2.0, and 1.5 were observed for the ZQG-azide, ZQG-alkyne, and ZQG-linker-cytotoxin respectively, all modified on the HC K447. In general, non-acidic, non-proline amino acids neighbouring K447 were found to improve transamidation efficiency. Engineering mAbs with multiple acceptor sites could increase DAR beyond 2.0, with a T135K-HC/L201K-LC proving to be a leading example with a DAR 4.0. However, it is worth noting that DARs could range between 0.8–5.0, depending on the ZQG-reagent used and the site of acyl acceptor insertion, with it difficult to predict the most appropriate sequences needed to engineer optimal insertion sites.

More recently, Bode *et al.* reported a chemoenzymatic method for lysine acylation which they called ‘lysine acylation using conjugation enzymes’ (LACE).^56^ This approach uses a small ubiquitin-like modifier (SUMO)-conjugating Ubc9 enzyme, which can identify an IKXE sequence on proteins, alongside a functionalised thioester as an acyl donor. This strategy enables the site-selective modification of the lysine residue within the IKXE tag. Initial work on 11-residue peptides containing an IKXE tag showed promise – 95% conversion into the lysine-modified product was obtained within short timeframes (4–8 h). Further investigation occurred on a one-pot site-selective dual modification strategy on trastuzumab Fab. Fab was engineered with the LACE tag IKSE on the LC, and a sortag LPETGG on the HC. Both insertions occurred on the constant domains of their respective chains. In the first step, standard conjugation conditions were employed, adding Ubc9, a rhodamine thioester, and a coumarin-modified triglycine, over 6 h at 30 °C. Then, Srt A was added, and the reaction was incubated over 4 h at 4 °C. Through this method, Fab was sequentially equipped with rhodamine, through the LACE tag using Ubc9, and coumarin, through the sortag using Srt A (Scheme 5c). Size-exclusion chromatography was used to purify the Fab conjugate, and subsequent analysis revealed clean, dual-modification of Fab. Using in-gel fluorescence, it was possible to confirm rhodamine modification occurred exclusively on the HC, selectively on the lysine of IKSE, while coumarin modification occurred exclusively on the LC. Additionally, HER2 binding affinity of the resultant Fab conjugate was retained, confirming the CDRs were unaffected by the modification. While only fluorophores were attached in this study, the strategy could potentially enable the modification of antibodies with two different cytotoxic drugs with complementary mechanisms of actions. This work further exemplifies the power of enzyme-directed modifications, as different enzymes can be used in combination with each other, enabling one-pot site-selective modifications of their respective recognition sites.

It is apparent that enzymes provide a very valuable contribution to the chemical biology toolbox for site-selective modification. While only a few reports are currently published on enzyme-directed approaches for site-selective lysine modification for ADC synthesis, there is certainly significant promise in the field. Enzymes offer the advantages of very high site-selectivity, conversions, and one-pot dual modification is achievable. However, there are some drawbacks to the use of enzymes – most notably that protein engineering is commonly required to ensure an appropriate recognition tag is present, increasing the complexity and costs involved with these strategies.

## 4. Proximity-induced site-selective lysine modification

As discussed, enzymes can achieve exquisite selectivity through proximity control, ensuring substrate reactivity occurs only in the active site. Mimicking nature, it is possible to apply a similar idea for selective lysine modification, using nearby structural motifs and functional groups to guide the labelling reaction. There are two predominant strategies of achieving proximity-induced modification – through affinity labelling, using peptide or small molecule binders to guide the reagents towards the intended modification site; or through covalent tethering, which utilises an initial, temporary covalent attachment site, before a second step enables modification of the target residue.

### 4.1. Affinity labelling

Affinity-guided conjugation strategies involve the incorporation of a scaffold that enables non-covalent interactions to occur between the affinity reagent and the target protein. This, in turn, increases local concentration of a reactive moiety to a particular site, allowing for site-specific modifications to take place. Affinity elements are often peptides with favourable interactions to a particular binding site, or small molecules that can localise to small pockets on the protein target. These affinity-guided reagents could then include, for example, non-canonical amino acids (ncAAs) which provide a bioorthogonal functionality that can be easily and selectively targeted, such as azides and alkynes, for purposes ranging from photoactivation to protein labelling.^57^

**Scheme 6:**
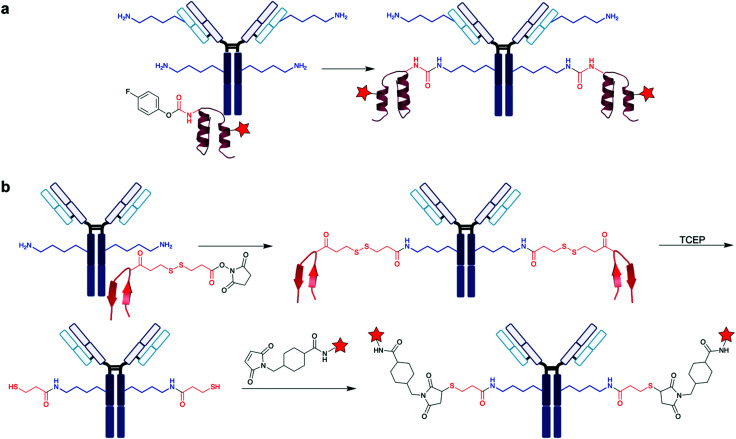
Examples of affinity-guided approaches for site-selective lysine modification, using peptides. Red star = functionality. (a) Xiao and co-workers protocol, utilising FB protein encoded with 4-fluorophenyl carbamate and a fluorophore. The FB protein binds in the Fc region of IgG.^58^ PDB 1FC2. (b) The AJICAP platform for homogeneous lysine-conjugated ADC preparation, developed by Mendelsohn *et al.*^61^ A similar affinity peptide to Ig-BP, discovered by Ito *et al.*, was used. PDB 6IQG.

Xiao *et al.* developed a ncAA-encoded affinity peptide-based protocol for the modification of lysine on IgG.^58^ Recognising the selectivity advantage of ncAAs, the group synthesised ncAA-containing peptides based on the B domain of protein A (FB protein) derived from *Staphylococcus aureus.*^59^ FB protein is a small, stable protein that only binds to IgGs in their Fc regions, preventing interference with the CDR or Fc receptor binding site of the antibody to preserve full antigen binding properties. Co-crystal structure analysis of a FB-trastuzumab complex revealed that there are several regions on FB with close proximity to a lysine residue on trastuzumab. This includes L18 and H19 of FB in close proximity to K316 on trastuzumab, and E25, E26, R28, and N29 on FB in close proximity to K337 on the antibody. Six FB protein mutants were developed, where the six residues found to be near a trastuzumab lysine were transformed into an electrophilic, ncAA 4-fluorophenyl carbamate lysine (FPheK), capable of cross-linking to form a stable urea (Scheme 6a). Only the E25FPheK mutant provided the desired FB-HC complex, in a 95% efficiency over 48 h at 37 °C, pH 8.5, with MS/MS sequencing confirming site-selective, single modification of K337, as expected from the co-crystal structure. The high efficiency and complete selectivity meant the E25FPheK mutant was further explored. Using a fluorophore-containing E25FPheK FB protein, a trastuzumab–fluorophore conjugate was generated in a 98% conversion. HER2 binding ability was retained too, with fluorescent imaging showing fluorescence only in HER2+ cells. Through understanding of protein–protein interfaces, Xiao *et al.* showed the use of affinity peptides to enable favourable positioning and proximity of amino acids, utilising lysine reactivity and electrophilic moieties for site-selective cross-linkages.

Other affinity peptides have also been explored for ADC synthesis, with these peptides often demonstrating high affinities for the Fc region of IgG. Ito *et al.* explored this further, developing a technique they termed chemical conjugation by affinity peptide (CCAP), starting with a 17-residue peptide, Ig-BP, from a random peptide library.^60^ This peptide is intramolecularly cross-linked *via* a disulfide bond, and binds to the Fc region of human IgG_1_ with a *K*_d_ of 10 nM. The group used co-crystal structure analysis of the Ig-BP-human IgG_1_-Fc complex to identify proximal interaction sites, finding the residue R8 of Ig-BP is within 10 Å of K248 on the HC of IgG_1_-Fc, a suitable distance for proximity-induced modification. The group replaced R8 with an inserted lysine residue (R8K) to develop Ig-BP/R8K. Using the cross-linking reagent disuccinimidyl glutarate, an NHS-Ig-BP–biotin peptide was then synthesised. 5 molar equivalents of the reagent was added to human IgG_1_ yielding ∼45% of the monovalent conjugate after 15 min at RT, in the pH range 4.5–7.0. After 1 h incubation at 22 °C, pH 7.0, only the divalent conjugate was present. With confidence in the efficiency of the Ig-BP/R8K peptide, the group moved onto executing the methodology on trastuzumab. In only 0.5 h at 22 °C, pH 5.5, divalently labelled trastuzumab was obtained, with peptide digestion and analysis confirming modification at K248. An ADC was then generated, first synthesising an NHS-Ig-BP-DM1 reagent, then following through CCAP. DARs of 1.0 and 2.0 were obtained for the monovalent and divalent ADCs respectively, while HER2 binding affinity was retained. In an anti-tumour proliferative assay with HER2+ SKBR-3 cells, the divalent ADC showed 3-fold increase in IC_50_ compared to Kadcyla (IC_50_ 50 pM), while the monovalent ADC demonstrated a similar IC_50_. Interestingly, Kadcyla has the highest DAR (3.5) of these ADCs, however, it is heterogeneously modified on various lysine residues. This work provides further evidence of the potential long-term clinical benefits of pursuing homogeneous lysine bioconjugation in ADC synthesis.

Using a similar IgG affinity peptide to that developed by Ito *et al.*, Mendelsohn and co-workers reported on the use of NHS esters in an affinity-mediated transfer (Scheme 6b). The group developed the affinity peptide mediated regiodivergent functionalisation (AJICAP) platform for the synthesis of ADCs from native IgG antibodies, which enables functionalisation of proximal lysine residues.^61^ Co-crystal structure analysis of the affinity peptides Fc-III and Z34C and the Fc region of human IgG identified a number of lysine residues with suitable proximity – Fc-III L6 and Fc K248 with a distance of 5.8 Å; and in Z34C, M3, R31 and E20 to Fc K248, K288, and K317 had approximate distances of 12.5, 13.7, and 4.0 Å respectively. Short affinity peptides containing a single lysine residue were modified with dithiobis(succinimidyl propionate), which contains a disulfide bond and two NHS ester moieties. These modified peptides were then added to trastuzumab. Promisingly, when targeting K246 and K248 using the novel affinity peptide, it was possible for Mendelsohn *et al.* to obtain 95% conversion in 1 h at 22 °C, pH 5.5, with a peptide-to-antibody ratio (PAR) of 2. Peptide digestion and sequencing *via* MS/MS confirmed modification at K246 and K248. As reported previously, K248 is the proximal lysine with a distance of only 5.8 Å to Fc-III L6, whereas K246 is significantly more distal, with a 17.3 Å distance to the same residue. It is likely therefore that the major modification occurs at K248. For affinity peptides based on Z34C targeting the same K246 and K248 residues, 91% conversion into PAR 2 was obtained, with small amounts of PAR 1 and PAR 3 species also present. The group did extensive work on developing multiple affinity peptides to target each individual proximal lysine within close proximity to the Fc-III and Z34C residues, finding desirable site-selectivity in all cases. The AJICAP platform was then applied for the synthesis of an ADC, using an affinity peptide with an installed cytotoxin DM1 through a thiol–maleimide reaction, following disulfide reduction. A DAR of 1.9 was achieved, with full retention of antigen binding. At 5 mg kg^−1^, the resultant ADC was able to demonstrate superior anti-tumour effects than trastuzumab at 20 mg kg^−1^. Promisingly, the AJICAP ADC exhibited a more sustained reduction in tumour volume over time, with no tumour growth observed over the course of the study. In comparison, with trastuzumab treatment, slow tumour growth was observed after one week, further exemplifying the potential therapeutic benefits of ADCs. In all of these bioconjugations, it was crucial for pH to be maintained at 5.5 to prevent unspecific conjugation and NHS ester hydrolysis.

**Scheme 7:**
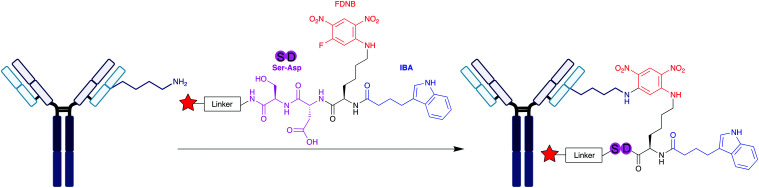
IBA-derivatised peptides for the labelling of lysine in the NBD on a mAb, developed by Lam *et al.*^64^ Pink circles = amino acids, blue circle = IBA, red star = functionality.

In the Fab region of all antibody isotypes, a highly conserved nucleotide binding domain (NBD) is located between the light and heavy domains of the antibody.^62^ Previous work conducted by Bilgicer and co-workers understood that indole-3-butyric acid (IBA) binds to this conserved pocket with high affinity (*K*_d_ = 1–8 μM), and as a result IBAs have become interesting reagents for selective loading at the NBD.^63^ This was explored more thoroughly on human mAbs by Lam *et al.*, who reported the synthesis and application of indole-based, 5-fluoro-2,4-dinitrobenzene (FDNB) derivatised peptides for site-specific introduction of bioorthogonal moieties (Scheme 7).^64^ A number of commercially available mAbs were explored, including bevacizumab, rituximab and trastuzumab. Crystal structure analysis of the Fab domain of trastuzumab identified that residues H101, H103, and L36 are capable of proximal interactions with IBA.^65^ Lysine residues near the NBD were chosen as targets, and following *in silico* docking results, the group designed indole-butyrate-Lys(FDNB)-derivatised dipeptides. Incorporating a negative charge through aspartic acid or glutamic acid on these dipeptides was found to improve affinity for the NBD lysines of trastuzumab. An affinity element containing aspartic acid and serine residues (DS peptide), with a biotin moiety, proved the most effective at NBD conjugation. Incubation of 5 molar equivalents of the reagent with trastuzumab at pH 8.5 (1 h, 22 °C) showed modification of the LC and HC when observed by western blot, corresponding to attachment at the NBD. Expanding onto three commercially available mAbs, the group observed consistency in their results – in all cases, following partial peptide digestion and western blot analysis, only the Fab fragment was modified by the reagent containing the affinity element, with no modifications seen in the Fc fragments, suggesting site-specificity for the NBD. On trastuzumab, a biotin–antibody-ratio of 1.6 was obtained when using the biotin DS peptide, compared to a biotin–antibody-ratio of 6.2 when using a biotin NHS ester. However, it was not possible to confirm site-selectivity for a single lysine residue, as there are several lysine residues neighbouring the NBD. Optimisation of reaction conditions identified 2.5 equivalents of the DS peptide would derivatise trastuzumab at pH 8.5 in 15 minutes at 22 °C. They lastly generated an ADC with this protocol, using a MMAE-derivatised DS peptide, conjugating the cytotoxin MMAE onto the lysines neighbouring the NBD of trastuzumab's Fab region – no loss of HER2 binding affinity in HER+ SKBR-3 cells was observed, and the ADC demonstrated potent cell-killing activity. This confirms that while not residue-specific, the site-selectivity towards the few lysine residues in the NBD does not confer an interference to the antigen-binding properties of the CDRs of the antibody.

**Scheme 8:**
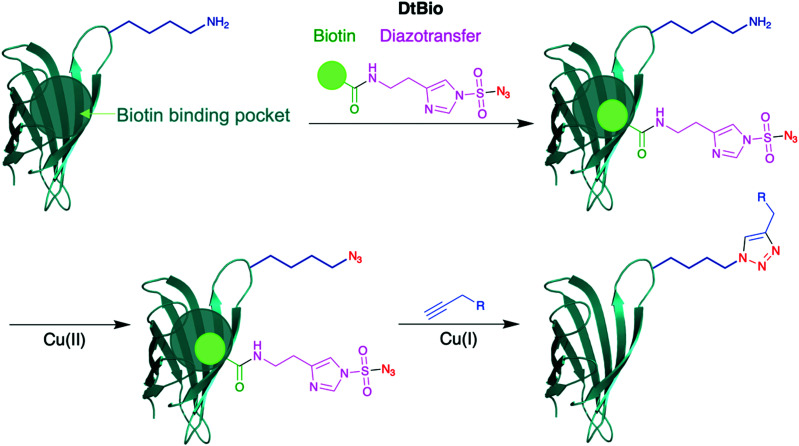
Structure of DtBio, consisting of a biotin directing group and a diazotransfer reagent, and its subsequent application to a lysine-containing peptide. Following diazotransfer to convert the amine group into an azide, click chemistry is possible to achieve site-selective labelling.^66^

An intriguing strategy for lysine modification, yet to be reported on antibodies, involves transforming the ε-amino sidechain of lysine directly into a bioorthogonal moiety. Diazotransfer reagents have been used for this purpose, as reported by Witte and co-workers (Scheme 8).^66^ They synthesised a biotin-tethered diazotransfer reagent, titled DtBio, which chemoselectively modifies lysine residues into azides. DtBio consists of biotin directing group which guides the reagent towards a particular binding site – for the purposes of their experiments, Witte *et al.* investigated DtBio on the biotin-binding protein streptavidin in the presence of ovalbumin, a competing protein. In this particular case, K121 is located near the biotin-binding site of streptavidin, close enough for potential proximity-induced site-selectivity. Post-conjugation conditions, DtBio was functionalised with a BODIPY-alkyne for fluorescent labelling *via* CuAAC. Fluorescence was detected only on K121-containing streptavidin, with none observed on ovalbumin, confirming selective diazotransfer onto only K121. The diazotransfer reaction is fast enough to occur even in the absence of copper catalyst, albeit at lower efficiencies. Enzymatic digestion and subsequence analysis *via* LC-MS/MS identified that only K121 was modified by DtBio, with no modification seen on K132 or K134. Thus, there is clear preference for the lysine located near the ligand binding site. A structural relative of streptavidin, avidin, contains a K111 residue in a very similar biotin-binding site – this residue also could be site-selectively modified by DtBio, further demonstrating the versatility of these diazotransfer reagents. This unconventional lysine modification strategy could prove of significant benefit to future bioconjugations, as it enables the insertion of bioorthogonal groups site-selectively for late-stage functionalisation. Instead of biotin localising to a biotin-binding region, it would be very interesting to observe whether other affinity peptide moieties, such as those used for IgG lysine modification, can enable a similar diazotransfer, which would significantly expand the usefulness of Witte and co-workers’ approach for other chemical biology applications. In particular, ADCs synthesised through this method could utilise click chemistry to directly attach cytotoxic payloads to the azide in a homogeneous manner.

### 4.2. Covalent tethering

Using covalent tethers, an initial, often reversible, reaction on a temporary binding site can bring another reactive moiety in closer proximity to the intended site of modification. Furthermore, it is possible to synthesise chemical reagents that can take advantage of the difference in reactivities between amino acid sidechains in different conditions, such as pH, and so it is possible to further tune selectivity for sites of modification. An example of covalent tethering in practice is native chemical ligation (NCL), which is commonly used for site-selective protein synthesis, utilising a thioester-mediated chemoselective reaction.^67^ Initially, a rate-determining reversible transthioesterification between a C-terminal thioester and N-terminal cysteine occurs, before a spontaneous *S*-to-*N* acyl transfer *via* a 5-membered ring intermediate irreversibly acylates a nearby amine to form an amide bond. This approach can also be applied to large, macrocyclic ring intermediates,^68^ with studies showing internal cysteines can help accelerate ligation onto the N-terminus.^69–72^

**Scheme 9:**
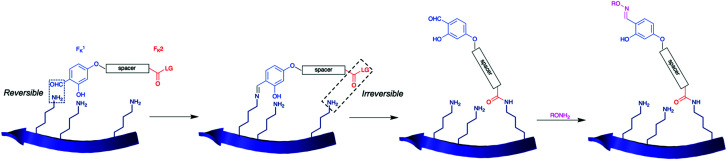
General scheme for the linchpin platform protocol developed by Rai *et al.* for the site-selective, proximity-controlled modification of lysine residues.^74^ An intermolecular, reversible, fast reaction with F^1^_K_ temporarily tethers the linchpin reagent in place, where an intramolecular, irreversible, slow reaction with F^2^_K_ labels a nearby lysine.

More recently, Rai *et al.* developed a ‘linchpin’ platform to achieve proximity-controlled site-selective modification of nucleophilic amino acid residues, first explored through histidine modification,^73^ before expanding the technique onto lysine residues.^74^ The method is based upon a linchpin compound, which features two chemoselective handles (Scheme 9). Whilst one handle undergoes a rapid, unselective, and reversible reaction with all solvent accessible lysine residues (F^1^_K_), the other handle reacts relatively slowly with lysine residues in an irreversible manner (F^2^_K_). Spacer design confers site-selectivity, as only lysine residues appropriately distanced from each other can undergo labelling. F^1^_K_ was selected as a *o*-hydroxybenzaldehyde core, which can reversibly and rapidly form imines upon lysine addition. To select suitable functional groups for F^2^_K_, the group screened various activated carbonyls, and investigated reactivity on RNase A (contains 10 lysine residues). Using a (3,5-dibromophenyl)(piperidin-1-yl)methanone leaving group at F^2^_K_ yielded the least heterogeneous labelling (7%) with low rates of hydrolysis; with F^1^_K_ and F^2^_K_ determined, the group developed various linchpins with distinct spacers linking the two moieties. Following bioconjugation, to remove excess linchpin and any leftover F^1^_K_–protein conjugates, *O*-hydroxylamine was added. Using a candidate linchpin, the group demonstrated labelling of RNase in a 34% conversion, with site-selective modification of K37, the most reactive lysine residue, observed through MS/MS sequencing. Rai *et al.* could then demonstrate selective labelling of a lysine in a library of proteins, though notably conversions were incomplete, ranging from 26–48% in ∼8 h. To increase functionality of their linchpin strategy, they also treated the aldehyde moiety of F^1^_K_ with functionalised *O*-hydroxylamine derivatives. The linchpin platform was next explored for ADC preparation. The acylation of K169 (LC) and K395 (HC) occurred over 16 h at 25 °C, pH 7.0. Subsequent functionalisation with DM1 was possible using a DM1-derivatised hydroxylamine, over 3 h at 25 °C. No conversion or DAR was reported for the synthesis of this trastuzumab-DM1 ADC. Modification was found to have occurred on K169 and K395, thus the linchpin showed selectivity for this lysine pair, over several others present in trastuzumab. The resultant ADC exhibited similar anti-tumour effects to that of Kadcyla against HER2+ SKBR-3 cells, with no impact on HER2− cells observed.

**Scheme 10:**
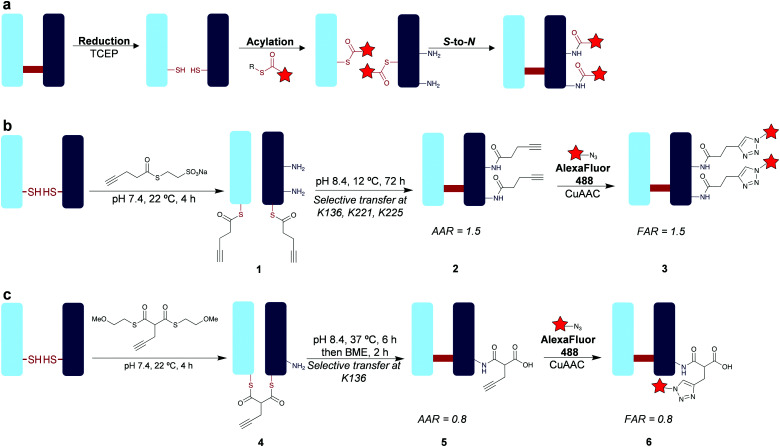
Investigations into the development of the CLT protocol.^75^ (a) General scheme of CLT. (b) CLT through the application of an alkyl thioester for labelling two lysine residues. (c) CLT through the application of a bis-thioester for the labelling of a single lysine. Red star = functionality (*e.g.* cytotoxin or fluorophore).

Recognising the merits and opportunities afforded by covalent tethering, we explored an approach inspired by NCL for lysine modification on a native antibody fragment through our cysteine-to-lysine transfer (CLT) protocol.^75^ Trastuzumab Fab is of widespread therapeutic interest for drug delivery and imaging applications,^76,77^ and consists of 26 lysine residues. Analysis of the crystal structure of Fab showed that there are three lysine residues on the heavy chain proximal to the single interchain disulfide bond (K136, K221, K225), and no proximal lysines on the LC. Importantly, these residues are also distal from the CDRs of Fab, which means modification should have no effect on the antigen-binding properties. We envisioned that it would be possible to use the cysteine residues composing the disulfide bond as temporary ligating ‘hooks’ to deliver an acylating agent to the nearby lysines through an *S*-to-*N* acyl transfer (Scheme 10a). To achieve this, we considered that thioesters present an ideal chemical reagent for CLT as they show a >100-fold increase in reactivity to amines than corresponding oxoesters whilst also being less reactive towards hydrolysis.^78^ Initial investigations using aryl thioesters on non-reduced Fab demonstrated a small amount of unselective lysine addition. We assumed that this is due, at least in part, to modification at K188; a very reactive lysine, supported by Cellitti's work on Fab using fluorophenyl esters.^32^ With a view to tuning down thioester reactivity and preventing this undesirable, non-specific reactivity, we next investigated alkyl thioesters. MESNa thioester was found to be completely inert upon reaction with non-reduced Fab even at 100 equivalents, while it undergoes highly selective transthioesterification with the cysteines in the reduced Fab under the same conditions to afford conjugate **1** (Scheme 10b). Having confirmed successful transthioesterification, we next explored the acyl transfer step to acylate the proximal lysine residues. We acknowledged the large macrocyclic intermediate involved in this CLT will likely slow the rate of acyl transfer significantly,^79^ thus we explored a range of conditions, measuring transfer completion to lysine amide conjugate **2** through the alkyne-to-antibody (AAR) ratio. Higher temperatures provided faster transfer reactions, but provided an AAR of 1.0, indicating significant amounts (∼50%) of competing hydrolysis. Optimal conditions of 12 °C, 72 h, pH 8.4 yielded complete transfer with an AAR of 1.5, representing 75% transfer. Treatment with excess thiols maintained the conjugate AAR of 1.5, indicating a robust and stable amide bond had formed chemoselectively on the proximal lysines. We could confirm regioselectivity on the HC too – reduction confirmed acylation exclusively on the HC. Tryptic digestion and LC-MS/MS analysis with 100% sequence coverage identified K136, K221, and K225 as the modification sites, as predicted from structure analysis. An ELISA confirmed complete retention of HER2 antigen binding, indicating the CDRs remain unaffected by our introduced modifications. We then generated Fab conjugate **3** using click chemistry to attach the fluorophore AlexaFluor488, with a fluorophore-to-antibody ratio (FAR) of 1.5.

To enable alternative stoichiometry of the CLT strategy, we explored the use of bis-thioesters (Scheme 10c). We postulated that the rigidity of the bridged system could further control regioselectivity, and the increased electron-withdrawing effect from the β-carbonyl could increase reactivity rates. Indeed, we observed the formation of the bridged conjugate **4** in just 30 minutes at 22 °C. Acyl transfer occurred optimally under conditions of 6 h, 37 °C, pH 8.4 to achieve the lysine-modified conjugate **5** with an AAR of 0.8, representing 80% transfer. Treatment with the thiol β-mercaptoethanol helped remove traces of mono-thioester formed through competing hydrolysis and confirmed stability as no reduction in AAR was observed. LC-MS/MS analysis showed selectivity for K136, and we could again generate a functionalised Fab conjugate **6** with matching FAR (0.8). Thus, we successfully demonstrated a proximity-induced, covalent tether strategy to achieve homogeneous, amide-forming, lysine-modified Fab conjugates. Such a strategy offers greatly improved homogeneity whilst still retaining the advantages of amide linkages. Additionally, CLT is very accessible – simple chemistry can be used to develop thioester reagents that can be easily applied on native mAbs, removing the technical complexity sometimes involved with the synthesis of, for example, peptides used in affinity-guided strategies, or antibodies engineered with specific conjugation sites.

The examples discussed here have outlined the immense potential and versatility of proximity-induced lysine modification. Methods to achieve proximity labelling of a target lysine range from affinity peptides with specificity for a particular binding site, to rationally designed small molecules utilising non-covalent interactions to a unique location, and finally covalent tethers which deliver reactive functional groups to specific residues. Overall, proximity-induced modifications offer the prospect of highly selective antibody labelling without the need for genetic engineering. However, the labelling using these approaches is currently limited in location by the site of binding of the affinity element or the site of attachment of the covalent tether. Further developments in available peptide (or other scaffold) affinity binders and covalent tethering can be anticipated to broaden the sites available for conjugation, which will provide a further level of control in the properties of the resultant bioconjugates.

## 5. Conclusion

Site-selective lysine modification is a key goal for bioconjugation research and is particularly sought for the construction of the next generation of antibody conjugates. In this feature article, we have outlined three principal strategies which have been described. Direct modification of a hyper-reactive lysine has the advantage of being a very straightforward process; however, high selectivity is very challenging to achieve and will be limited to specific proteins. Enzymatic modifications can offer very impressive selectivity, but commonly require engineering to insert a recognition tag, or specifically accessible substrate lysines. Finally, proximity-induced modification is a very promising approach, and we have discussed multiple examples of smartly designed peptides and small molecules that can take full advantage of potential affinity sites or covalent ‘hooks’. Our recently developed CLT method is an example of this, utilising cysteine residues as temporary ligating tethers and an intramolecular *S*-to-*N* transfer to achieve site-selective acylation of proximal lysine residues. This presents an easily accessible route to the synthesis of lysine-conjugated antibody structures with much improved homogeneity, using readily available thioester reagents. This CLT method can be further developed going forward, with new reagents, optimised conjugation efficiency and controlled modification of full antibodies, allowing an accessible approach to site-selective conjugates from native antibodies; which in-turn could improve the *in vivo* effectiveness of synthesised ADCs.

The development of new site-selective lysine bioconjugation strategies continue to be sought, as the ability to control the exact lysine which is modified, and doing so in high conversions, will allow additional control of the properties of the ADCs; which will be crucial to the future success of this therapeutic class. Such approaches should continue to be comprehensively characterised, to show that certain lysines have been modified selectively, which is a challenging task. We would suggest that intact protein LCMS (both the raw and deconvoluted data analysed) and LC-MS/MS are used as the cornerstones. The former provides evidence for the conversions and number of attachments to the protein, and the latter reveals the exact residues modified. For antibodies, and antibody fragments, reduction of the interchain disulfides followed by capping (*e.g.* with maleimide) and subsequent LCMS analysis provides convenient additional evidence of selective modification of either the LC or HC. SDS-PAGE and analysis of the attached functionality (*e.g.* UV absorbance if the attached drug contains a chromophore) can provide further valuable supporting evidence.

Enticingly the prospect of combining multiple lysine selective bioconjugation strategies together can be envisaged, which could enable new designs and applications of ADCs. For example, by allowing the controlled addition of multiple different drugs to an antibody with complimentary modes of action to overcome resistance mechanisms. Such methods to enable the attachment of multiple functionalities (*e.g.* drugs, imaging agents, cross-linking of multiple antibody fragments to create bispecifics etc) in a site-selective manner *via* robustly stable linkages to lysine is likely to represent key enabling technologies for the next generation of ADCs and related targeted therapeutics and diagnostics.

## Conflicts of interest

There are no conflicts to declare.

## Supplementary Material
